# Groups of Symmetries of the Two Classes of Synthetases in the Four-Dimensional Hypercubes of the Extended Code Type II

**DOI:** 10.3390/life13102002

**Published:** 2023-09-30

**Authors:** Marco V. José, Eberto R. Morgado, Juan R. Bobadilla

**Affiliations:** 1 Theoretical Biology Group, Instituto de Investigaciones Biomédicas, Universidad Nacional Autónoma de México, Ciudad de México 04510, Mexico; jromanb@unam.mx; 2 Facultad de Matemática, Física y Computación, Universidad Central “Marta Abreu” de Las Villas, Santa Clara 50100, Cuba; ebertorodobaldo@gmail.com

**Keywords:** aminoacyl-tRNA synthetases, standard genetic code, symmetry groups, origin and evolution of the genetic code, group theory

## Abstract

Aminoacyl-tRNA synthetases (aaRSs) originated from an ancestral bidirectional gene (mirror symmetry), and through the evolution of the genetic code, the twenty aaRSs exhibit a symmetrical distribution in a 6-dimensional hypercube of the Standard Genetic Code. In this work, we assume a primeval RNY code and the Extended Genetic RNA code type II, which includes codons of the types YNY, YNR, and RNR. Each of the four subsets of codons can be represented in a 4-dimensional hypercube. Altogether, these 4 subcodes constitute the 6-dimensional representation of the SGC. We identify the aaRSs symmetry groups in each of these hypercubes. We show that each of the four hypercubes contains the following sets of symmetries for the two known Classes of synthetases: RNY: dihedral group of order 4; YNY: binary group; YNR: amplified octahedral group; and RNR: binary group. We demonstrate that for each hypercube, the group of symmetries in Class 1 is the same as the group of symmetries in Class 2. The biological implications of these findings are discussed.

## 1. Introduction

Aminoacyl-tRNA synthetases (aaRSs) are key players in the genetic code of all living beings. AaRS attaches an amino acid to the cognate tRNA, and the aminoacyl-tRNA is then used for translation upon binding to mRNA according to the codon-anticodon interaction on the ribosome. The Standard Genetic Code (SGC) is the mapping of 61 codons or triplets to 20 canonical amino acids. There are 20 aaRSs, one for each of the 20 standard amino acids. AaRSs are divided into two mutually exclusive Classes, 1 and 2, based on their structural, functional, and evolutionary relatedness [1,2,3,4,5,6,7,8]. Each aaRS falls into either Class 1 or Class 2, except for lysyl-tRNA synthetase (LysRS), which has a representative in both classes. The correspondence between amino acids and synthetases is one-to-one; that is, it is a bijective, non-degenerate code. For every triplet, or codon, there is a synthetase associated with the amino acid it specifies. In a previous work [9], we outlined the group of symmetries of both Classes of synthetases in each of the 4-dimensional hypercubes of the so-called Extended Code of type II [10,11]. Herein, we rigorously determine the symmetries of aaRSs in the 4 subcodes RNY, YNY, YNR, and RNR. The article is organized as follows: First, a succinct description of the Table of the genetic code, highlighting the subcodes and the Classes of aaRSs, Next, the arithmetization of the SGC is introduced. Second, we provide the basic mathematical concepts needed to understand the derivation of the symmetries of the aaRSs in the 4 subcodes and in the whole SGC. In Appendix E, we present formal mathematical definitions and concepts. Third, we determine the symmetries in each of the four subcodes, where the mathematical derivations are explained in detail in a series of four Appendixes. We prove that the group of symmetries of both classes of synthetases in the hypercube RNY, is the dihedral group $D_{4};$ the group of symmetries of both Classes of synthetases in the hypercube YNY is the binary group.$\left(Z_{2},{⊕}_{2}\right);$ The group of symmetries of both classes of synthetases in the hypercube YNR is the extended octahedral group $\left(O_{h},\circ \right);$ and the group of symmetries of the Classes of synthetases in the hypercube RNR is the group $\left(\left\{R,I\right\}\circ \right),$ isomorphic to the Abelian group $\left(Z_{2},{⊕}_{2}\right),$ where the reflection R is the only symmetry of both Classes 1 and 2, and I is the identity matrix.

Notably, we show that the group of symmetries in Class 1 is the same as the group of symmetries in Class 2 for each of the 4 subcodes. The 4 subcodes constitute the whole SGC, which displays a mirror symmetry of aaRSs. We also analyzed the 5-dimensional hypercubes NNY (all triplets that end in pyrimidine) and NNR (all triplets that end in purine) as obtained by the union of RNY with YNY and YNR with RNR, respectively. We remark that the latter step has been simply ignored in the evolution of the SGC. Finally, we discuss the biological implications of the results, in terms of the evolution of the SGC and in how the code went from managing the information by quadruples of 0’s and 1’s to sextuples of 0’1 and 1’s, where the distributions of aaRSs in the subcodes obeyed symmetrical groups.

### 1.1. Basic Mathematics of the SGC

The cartesian product $N^{3}=N\times N\times N,$ being $N=\left\{C,U,A,G\right\}$ the set of the four RNA-nucleotides, C = Cytosine, U = Uracil, A = Adenine, and G = Guanine, is the set of the 64 triplets $\left(X,Y,Z\right),$ where X,Y,Z∈N. The standard genetic code (SGC) may be seen as a surjective function $f:N^{3}\to A_{s}=A\cup \left\{S\right\},$ being A the set of the 20 known amino acids, and S the stop signal, which means the instruction of finalizing the process of synthesis of a protein.

For every a∈A, preimage set $f^{-1}\left\{a\right\}=\left\{\left(X,Y,Z\right)\left|f(X,Y,Z)=a\right.\right\},$ is the set of triplets, also called codons, that encode the amino acid, or stop signal, $a\in A_{s}.$ Since the decipherment of the genetic code [12], it is known that the number of coding triplets for any $a\in A_{s}$ is one of the numbers 1, 2, 3, 4, or 6.

In Table 1, we show the table of the genetic code, highlighting the 4 subcodes RNY, YNY, YNR, and RNR, as well as the 2 Classes of synthetases. The table also indicates the subcodes NNY and NNR.

### 1.2. Arithmetization of the Genetic Code

The bijective correspondence C↔00, U↔01, A↔10,G↔11, between the set N and the set $Z_{2}^{2}=Z_{2}\times Z_{2},$ being $Z_{2}=\left\{0,1\right\},$ induces a bijective correspondence between the set NNN of the 64 triplets and the set $Z_{2}^{6}$ of all the sextuples of zeros and ones.

Binary operations: In the binary set $Z_{2}=\left\{0,1\right\},$ the two binary operations ${⊕}_{2},{⊗}_{2},$ the so-called addition and product, module 2, are defined. Their Cayley Tables (Table 2) are:

They define in the set $Z_{2}=\left\{0,1\right\},$ the algebraic structure of a field, or commutative division ring. This is the smallest possible field, with only two elements: the neutral of the addition and the neutral of the product, respectively.

The addition ${⊕}_{2}$ is extended, component-wise, to the set $Z_{2}^{6},$ which, with the natural definition of the product of any scalar $\alpha \in Z_{2}$, by a sextuple $\left(a_{1},a_{2},a_{3},a_{4},a_{5},a_{6}\right),$ becomes a 6-dimensional vector space over the binary field O=(0,0,0,0). This vector space is the so-called 6-dimensional binary hypercube.

The bijection between $Z_{2}^{6}$ and the set NNN of the 64 triplets, induces in it the algebraic structure of a 6-dimensional vector space, whose canonical basis is the system: (ACC, UCC, CAC, CUC, CCA, CCU). The addition of nucleotides is shown in the following Table 3:

It is seen that the nucleotide C, cytosine, is the neutral element of the group. This group is isomorphic to the de group, $\left(Z_{2},Z_{2},⊕\right),$ which is known as the Four Klein Group. It is an Abelian group of order 4, where each element has its own inverse. It is, in the Felix Klein list of finite groups, the fourth and first that is not cyclic.

**Mutations in the triplets of the genetic code:** A mutation in a triplet XYZ is the substitution of any of its components by a nucleotide. For example, GUC→GAC, where U is replaced by A, Obviously, a mutation in a triplet may produce a change in the amino acid it encodes. In our example, valine, which is encoded by GUC, is converted into aspartic acid, encoded by GAC. Algebraically, a mutation in a triplet is performed by the addition of triplets to the canonical basis. In our example, the mutation GUC→GAC is obtained by the addition of the triplet CGC = CAC + CUC.

**Classification of the nucleotides**

There is a partition $\left\{Y,R\right\}$ of the set N, being $Y=\left\{C,U\right\}$ pyrimidines and $R=\left\{A,G\right\}$ purines. The condition of being a pyrimidine or a purine is called the chemical type of a nucleotide.

**Transitions and transversion**: A mutation is called a transition if it does not change the chemical type, and it is called a transversion if the chemical type is changed. It is easy to notice that transitions are produced by the addition of pyrimidines and transversions by the addition of purines.

**The primaeval genetic code**. It is assumed that there was, in the RNA world, a primitive, or primaeval, genetic code of only sixteen triplets and eight amino acids: the set $\mathrm{RNY}=\left\{\left(X,Y,Z\right)\right\},$ where X is a purine, Y is any of them, and Z is a pyrimidine. It is a 4-dimensional hypercube, a translation of the 4-dimensional subspace YNY, derived from RNY the transversion in the first nucleotide. By transversions in the first or third component, the hypercubes YNY,YNR and RNR are derived from the primaeval RNY. These four sets are pairwise disjoint, and they cover the whole set. NNN. Hence, the set of sets ${\left\{-1,1\right\}}^{4}$ is a partition of NNN. The hypercube YNY determines, with the addition ${⊕}_{2},$ a subgroup of the additive group $\left(\mathrm{NNN},{⊕}_{2}\right)$, the other RNY,YNR and RNR its cosets. With the addition and the product of scalars by vectors, YNY a 4-dimensional vector subspace RNY,YNR is determined, as RNR are its affine subspaces, that is, translations of it, namely:

$$\begin{array}{l} \mathrm{RNY}=YNY+\left\{\mathrm{ACC}\right\}\\ \mathrm{YNR}=YNY+\left\{\mathrm{CCA}\right\}\\ \mathrm{RNR}=YNY+\left\{\mathrm{ACA}\right\} \end{array}$$

### 1.3. The Classes of Synthetases

Synthetases are enzymes that regulate the selection of amino acids that will be charged to tRNA molecules. A little more than 20 aaRSs are found in modern organisms. They are classified into two groups, Class 1 and Class 2, each having three subclasses (a, b, and c) based on similarity in sequences and structures [13,14]. The classification is shown in Table 4:

In general, aaRS consists of a catalytic domain, an anticodon-binding domain, and often an editing domain. Each class harbors class-specific characteristic motifs and structural topology in its catalytic domains [3].

According to the RO model [15,16,17,18] the table of the genetic code can be divided into the sub-codes NAN, NGN, NUN, and NCN. We have also shown that there exists an automorphism *F* of the cube defined piecewise, which transforms that division into the sub-codes RNR, YNR, RNY, and YNY, respectively, which is precisely our algebraic model [19].

## 2. Results

### 2.1. Group of Symmetries of the Classes of Synthetases in the Hypercube RNY

**Figure 1:**
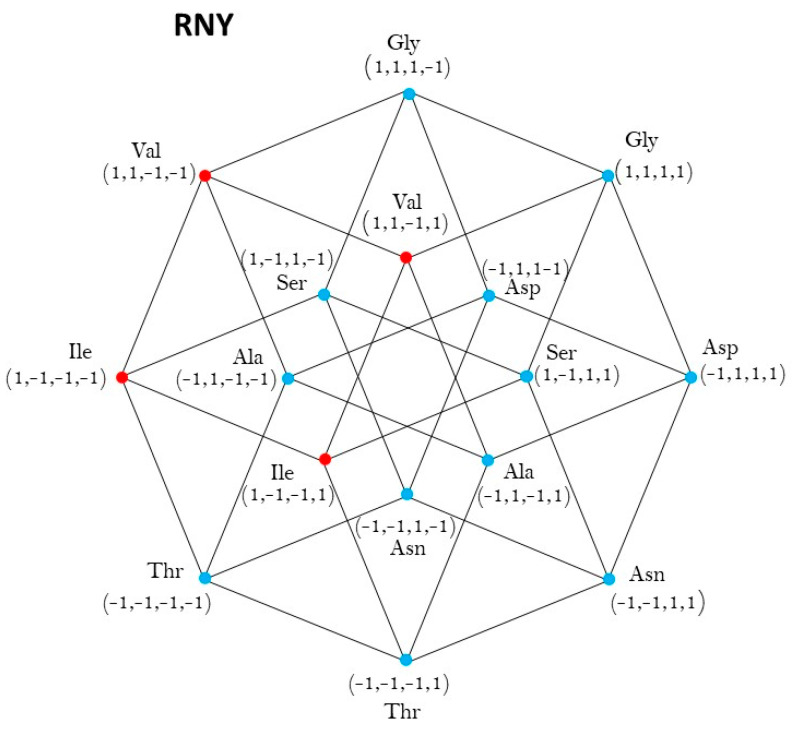
aaRSs distribution in codes RNY. Class 1 (red, 2 amino acids) and Class 2 (blue, 6 amino acids). Graphic representation of the subsets RYY and RRY. The first 4-dimensional hypercube of the RNY code: RNY=RYY∪RYY.

The members of Class 2 are in the vertices of a square, contained in the ${\left[-1,1\right]}^{4},$ convex closure of the set ${\left\{-1,1\right\}}^{4},$ taken by a suitable change of coordinates, rotations, and translations of axis as a representation of the hypercube RNY. See Figure 1:

The quadruplets that belong to Class 2 of synthetases (colored black) are in the set $V_{2}$ of vertexes of a square S, on one of the 24 faces of the hypercube. $V_{2}$ is a subset of the set ${\left\{-1,1\right\}}^{4}$ of the sixteen vertices of the hypercube RNY. The set of vertexes of that square is:

$$V_{2}=\left\{v_{1},v_{2},v_{3},v_{4}\right\},$$

where

v_1_ = (1,−1,−1,−1), v_2_ = (1,−1,−1,1), v_3_ = (1,1,−1,1), v_4_ = (1,1,−1,1), black colored.

The center of this square is the point $c_{2}=\frac{v_{1}+v_{2}+v_{3}+v_{4}}{4}=\left(1,0,-1,0\right)$. 

The members of Class 1 are in the complementary set of vertexes $V_{1},$ that is, the other 12 vertexes, red colored, out of the four vertexes of the square S.

v_1_ = (u_1_,u_2_,u_3_,u_4_,u_5_,u_6_,u_7_,u_8_,u_9_,u_10_,u_11_,u_12_) where

u_1_ = (−1,−1,−1,−1), u_2_ = (−1,−1,−1,1), u_3_ = (−1,1,−1,1), u_4_ = (−1,1,−1,−1)

u_5_ = (−1,−1,−1,−1), u_6_ = (−1,−1,1,1), u_7_ = (−1,1,1,1), u_8_ = (−1,1,1,−1)

u_9_ = (1,−1,1,−1), u_10_ = (1,−1,1,1), u_11_ = (1,1,1,1), u_12_ = (1,1,1,−1)

The main result of this Section 2.1 is: We have proved that the group of symmetries of both classes of synthetases, in the hypercube RNY, is the dihedral group $D_{4}.$ Details are given in Appendix A.

Now we can state the following:

**Theorem** **1.**  *The group of symmetries of Class 1 are the same as that of Class 2.*

**Proof.**  As the symmetry of Class 1 is a bijective isometrical function f from RNY onto itself, it preserves the set Class 1∈RNY. It also preserves the square S, that contains the quadruples of Class 2, that is, the vertices of the square S. It means that both classes have the same group of symmetries. That is so because the binary set of sets $\left\{\mathrm{Class}\;1,\;\mathrm{Class}\;2\right\}$ is a partition of the finite set ${\left\{-1,1\right\}}^{4}$ of the 16 vertices of the hypercube RNY.

Observation: A similar theorem takes place in any of the other hypercubes of Extended code type II and in the whole 6-dimensional hypercubes. NNN. In the case of the 4-dimensional YNR, the 5-dimensional NNR, and the whole 6-dimensional, NNN, it is valid if, for methodological reasons, we assume the stop signal as it would be an amino acid of Class 1.

### 2.2. Group of Symmetries of the Classes of Synthetases in the Hypercube YNY

**Figure 2:**
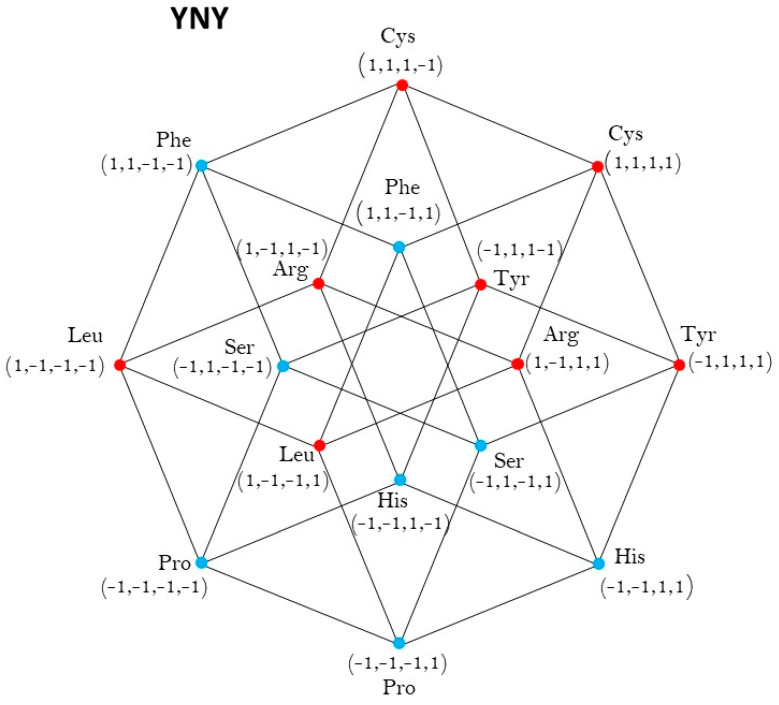
aaRSs distribution in the YNY code. Class 1 (red, 4 amino acids) and Class 2 (blue, 4 amino acids). Graphic representation of the subsets YYY and YRY. Second 4-dimensional hypercube of the Extended RNA-code type 2: YNY=YYY∪YRY.

In this case, the members of each class are in eight vertexes, out of the sixteen vertexes of the hypercube YNY (see Figure 2). They are represented by the sixteen quadruples of the set ${\left\{-1,1\right\}}^{4}$ of ones and minus one, being the hypercube ${\left[-1,1\right]}^{4},$ the convex closure of the set ${\left\{-1,1\right\}}^{4}.$ The set ${\left[-1,1\right]}^{4}$ is taken, by a suitable change of coordinates, rotations, and translations of axis, as a representation of the hypercube YNY. The center of this hypercube is the origin of coordinates O=(0,0,0,0).

The set of vertexes of the Class 1, colored in red, is the set:

$$V_{1}=\left\{\left(1,1,-1,-1\right),\left(1,1,-1,1\right),\left(-1,1,-1,-1\right),\left(-1,1,-1,1\right),\left(-1,-1,-1,-1\right),\left(-1,-1,-1,1\right),\left(-1,-1,1,-1\right),\left(-1,-1,1,1\right)\right\}.$$

The members of Class 2 are in the complementary set of vertexes, that is, the other eight vertexes, colored black, out of the eight of Class 1.

This is the set:

$$V_{2}=\left\{\left(1,-1,-1,-1\right),\left(1,-1,-1,1\right),\left(1,-1,1,-1\right),\left(1,-1,1,1\right),\left(1,1,1,-1\right),\left(1,1,1,1\right),\left(-1,1,1,-1\right),\left(-1,1,1,1\right)\right\}.$$

Let us consider the affine transformation: $T_{\left(1/2,1/2,1/2,1/2\right)}\circ \left(1/2\right)I,$ that converts each −1 into 0 and 1 into itself. It represents a change of coordinates, that converts the hypercube ${\left[-1,1\right]}^{4}$ into its isometric ${\left[0,1\right]}^{4},$ whose center is the point (1/2, 1/2, 1/2, 1/2). The sets $V_{1}$ and $V_{2}$ are now:

$$V_{1}=\left\{\left(1,1,0,0\right),\left(1,1,0,1\right),\left(0,1,0,0\right),\left(0,1,0,1\right),\left(0,0,0,0\right),\left(0,0,0,1\right),\left(0,0,1,0\right),\left(0,0,1,1\right)\right\}.$$

$$V_{2}=\left\{\left(1,0,0,0\right),\left(1,0,0,1\right),\left(1,0,1,0\right),\left(1,0,1,1\right),\left(1,1,1,0\right),\left(1,1,1,1\right),\left(0,1,1,0\right),\left(0,1,1,1\right)\right\}.$$

Then, the group of symmetries of both Classes of synthetases in the hypercube YNY is the binary group $\left(Z_{2},{⊕}_{2}\right);$ Details can be found in Appendix B.

### 2.3. Group of Symmetries of the Synthetases in the Hypercube YNR

**Figure 3:**
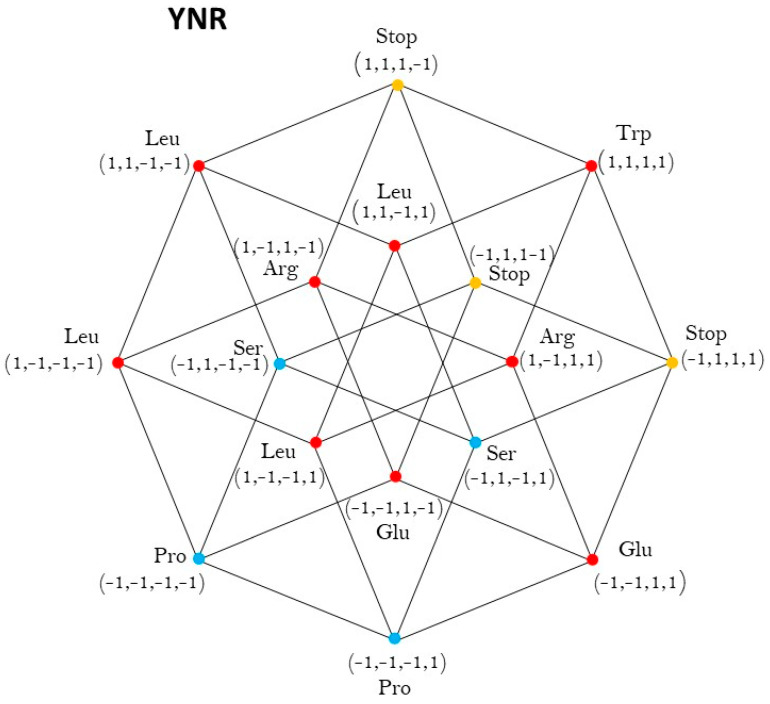
aaRSs distribution in the YNR code. Class 1 (red, 3 amino acids) and Class 2 (blue, 3 amino acids and yellow, the stop signals). Graphical representation of the subsets YYR and YRR. Third 4-dimensional hypercube of the Extended RNA− code type II: YNR=YYR∪YRR.

In this case there are 3 amino acids of Class 1 and 3 amino acids of Class 2 (Figure 3).

For methodological reasons, we have assumed the three stop codons are members of Class 1. In this case, the members of each Class are on the eight vertices of a 3-dimensional cube. Both Classes coincide with the cubes YNU and YNG. The vertices YNR are represented by the sixteen quadruples of the set ${\left\{-1,1\right\}}^{4}$ of ones and minus ones, being the hypercube ${\left[-1,1\right]}^{4},$ the convex closure of the set ${\left\{-1,1\right\}}^{4},$ The set ${\left[-1,1\right]}^{4}$ is taken, by a suitable change of coordinates, rotation, and translations of axis, as a representation of the hypercube YNR. The center of this hypercube is the origin of the coordinates. O=(0,0,0,0).

The set of vertexes of the cube, associated to Class I, colored in red, is the set:

$$V_{1}=\left\{\left(-1,-1,-1,-1\right),\left(-1,-1,-1,1\right),\left(-1,1,-1,1\right),\left(-1,1,-1,-1\right),\left(1,-1,-1,-1\right),\left(1,-1,-1,1\right),\left(1,1,-1,1\right),\left(1,1,-1,-1\right)\right\}.$$

The center of this cube is the point $-e_{1}=\left(0,0,-1,0\right),$ and the set of vertexes of the Class 2, colored in black, is the set:

$$V_{2}=\left\{\left(-1,-1,1,-1\right),\left(-1,-1,1,1\right),\left(-1,1,1,1\right),\left(-1,1,1,-1\right),\left(1,-1,1,-1\right),\left(1,-1,1,1\right),\left(1,1,1,1\right),\left(1,1,1,-1\right)\right\}.$$

whose center is the point $e_{3}=\left(0,0,1,0\right).$

Then, we have proved that the group of symmetries of both classes of synthetases in the hypercube YNR is the extended octahedral group $\left(O_{h},\circ \right).$ See Appendix C.

### 2.4. Group of Symmetries of the Synthetases in the Hypercube RNR

**Figure 4:**
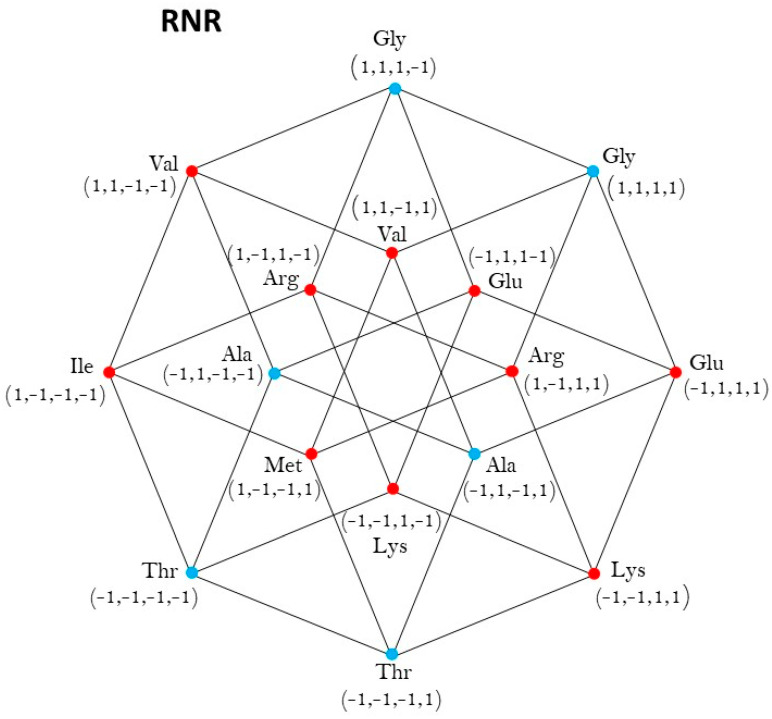
aaRSs distribution in the RNR code. Class I (red, 3 amino acids) and Class 2 (blue, 5 amino acids). Graphic representation of the subsets RYR and RRR. Fourth 4-dimensional hypercube of the Extended RNA-code type II: RNR=RYR∪RRR.

In this case the vertexes of Class 1, colored in red, are the set of six vectors (Figure 4):

$$V_{1}=\left\{v_{1}=\left(-1,-1,-1,-1\right),v_{2}=\left(-1,-1,-1,1\right),v_{3}=\left(-1,1,-1,1\right),v_{4}=\left(-1,1,-1,-1\right),v_{5}=\left(1,1,1,-1\right),v_{6}=\left(1,1,1,1\right)\right\},$$

which is the union of the square of vertexes $V_{1},V_{2},V_{3},V_{4}$ with the edge of vertexes $V_{5},V_{6}.$

And the set of vertexes of Class 2, colored in black, are the set of the ten vertexes (Figure 4):

$$V_{2}=\left\{\begin{array}{l} u_{1}=\left(1,-1,-1,-1\right),\;u_{2}=\left(1,-1,-1,1\right),\;u_{3}=\left(1,1,-1,1\right),\;u_{4}=\left(1,1,-1,-1\right),\;\\ u_{5}=\left(-1,-1,1,-1\right),\;u_{6}=\left(-1,-1,1,1\right),\;u_{7}=\left(-1,1,1,1\right),\;u_{8}=\left(-1,1,1,-1\right),\\ u_{9}=\left(1,-1,1,-1\right),\;u_{10}=\left(1,-1,1,1\right) \end{array}\right\}$$

which is he union of the squares of vertexes $u_{1},u_{2},u_{3},u_{4}$ and $u_{5},u_{6},u_{7},u_{8}$ with the edge of vertexes $u_{9},u_{10}.$

The group of symmetries of the Classes of synthetases in the hypercube RNR is $\left(\left\{R,I\right\}\circ \right),$ isomorphic to the Abelian group $\left(Z_{2},{⊕}_{2}\right),$ where the reflection R is the only symmetry of both Classes 1 and 2, and I is the identity matrix. A detailed derivation is given in Appendix D.

### 2.5. Conclusions

It has been proven that the groups of symmetries of both classes of synthetases in each of the four hypercubes are the following (Table 5):

## 3. The Genetic Code in 5 and 6 Dimensions

### 3.1. The Genetic 5-Dimensional Hypercube

The 5-dimensional hypercube NNY is the disjoint union of the 4-dimensional hypercubes YNY and RNY. YNY is the subspace generated by the four unitary vectors UCC, CAC, CUC, CCU and RNY is its affine subspace $\mathrm{YNY}+\left\{\mathrm{ACC}\right\}.$ Here, the sextuples of zeros and ones have been replaced by triplets of the letters C,U,A,G, with the following equivalence: C=00, U=01, A=10, G=11. Y is the binary set $\left\{C,U\right\},$ R is the also binary $\left\{A,G\right\},$ and N is the set $\left\{C,U,A,G\right\}=\left\{Y,R\right\}.$

**Figure 5:**
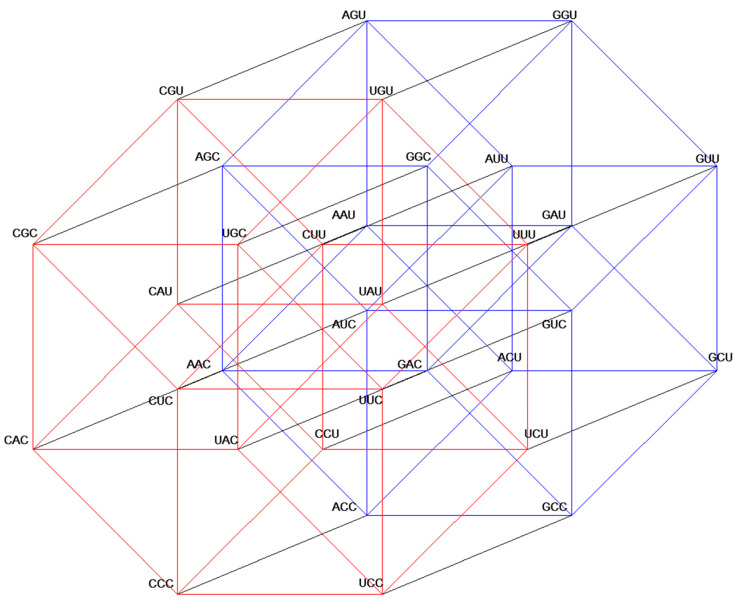
The five-dimensional hypercube of triplets NNY, which is the union of the 4-dimensional hypercubes RNY and YNY. YNY is obtained from the RNY by means of transversion of each nucleotide at the left of the triplet. YNY is the subspace generated by the four unitary vectors CAC, CUC, CCU, UCC, and RNY is its affine subspace $\mathrm{YNY}+\left\{\mathrm{ACC}\right\}.$ The translation that represents these transversions is situated at an angle of 22.5 degrees with respect to the horizontal axis generated by the canonical vector UCC. At this angle, none of the vertices are overlapped.

The four triplets UCC, CAC, CUC, CCU are associated with the canonical unitary vectors $e_{2},\;e_{3},\;e_{4}.\;e_{6}.$ In Figure 5, they are vectors initiated at the point CCC, colored red that generate the hypercube YNY. The triplet ACC, associated with the unitary vector $e_{1},$ also initiated CCC, and colored blue, completes the canonical basis of the 5-dimensional hypercube NNY. Obviously, the translation associated with converting YNY into its affine subspace RNY, is colored blue, and the union of both YNY with RNY, is the 5-dimensional hypercube NNY. The vertexes NNY are the triplets or codons that end in a pyrimidine.

**Figure A1:**
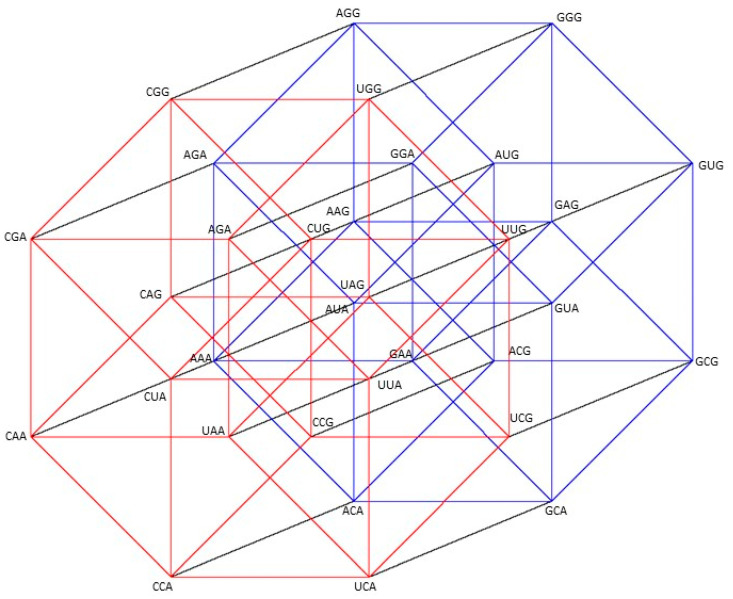
Five-dimensional hypercube NNR. The five-dimensional hypercube of triplets NNR, which is the union of the 4-dimensional hypercubes YNR and RNR.

It can be seen that the translation $T_{e},$ associated with the unitary vector $e_{5}$ represented by the triplet CCA, converts the 5-dimensional subspace NNY into its 5-dimensional affine subspace NNR, whose triplets are those that end in a purine (Figure A1). It is the disjoint union of the 4-dimensional hypercubes YNR and RNR. It is clear that the 6-dimensional hypercube NNN is the union of the 5-dimensional hypercube NNY with its affine subspace, also a 5-dimensional hypercube, $\mathrm{NNR}=\mathrm{NNY}+\left\{\mathrm{CCA}\right\}.$ Finally, we have, that NNN is the disjoint union RNY∪YNY∪YNR∪RNR of the four 4-dimensional hypercubes, derived from the primaeval RNY by transversions in the first or third nucleotide.

Note: The subspace (hypercube) YNY, is the solution set of the homogeneous system of two liner equations $x_{1}=0,\;x_{5}=0,$ with the six unknowns $x_{1},x_{2},x_{3},x_{4},x_{5}.x_{6}.$ Its affine subspace (hypercube) RNY, is the solution set of the non-homogeneous system $x_{1}=0,\;x_{5}=0,$ also of rank 2, with the same six unknowns.

The 5D hypercube NNR (see Figure A1) is obtained by the translation $T_{\mathrm{CCA}},$ that converts every triplet that ends in pyrimidine into another that ends in purine, obtaining the hypercube NNR. It represents the disjoint union of the subcodes YNR and RNR. Note that this hypercube contains the stop codons UGA,UAG and UAA. The disjoint union of NNY them, which NNR is equal to the 6D hypercube NNN that represents the entire SGC. For the 6D genetic code, each triplet is mapped into sextuples of o’s and 1's. This hypercube is the set NNN isomorphic to $Z_{2}^{6},$ which is the binary vector space over the binary field $Z_{2},$

### 3.2. The Hypercube of Dimension 6 NNN

It is the disjoint union of the 5-dimensional NNY space and NNR. NNY is the subspace generated by the five unitary vectors CAC, CUC, CCU, UCC, ACC, and NNR its affine subspace $\mathrm{NNY}+\left\{\mathrm{CCA}\right\}.$ The vector space NNN is generated by the six unitary vectors CAC, CUC, CCU, UCC, ACC, and CCA. They conform to the canonical basis of $Z_{2}^{6}=\mathrm{NNN}.$ The conversion of the hypercube ${\left[0,1\right]}^{n}$ in other whose components are ones and minus ones. Multiplying by 2 all the vectors of the space, the set of the $2^{n}$ vertexes is converted into the set ${\left\{0,2\right\}}^{n},$ of vertexes of the hypercube ${\left[0,2\right]}^{n}$ whose edges have length 2. Next, performing the substraction of the n-tuple $E=\left(1,1,\ldots ,1\right)$ the hypercube ${\left\{0,2\right\}}^{n}$ is converted into ${\left[-1,1\right]}^{n},$ which is another hypercube, also with edges of length 2, whose set of vertexes is the set ${\left\{-1,1\right\}}^{n}$ of the $2^{n}$ n-tuples of ones and minus ones.

**Figure 6:**
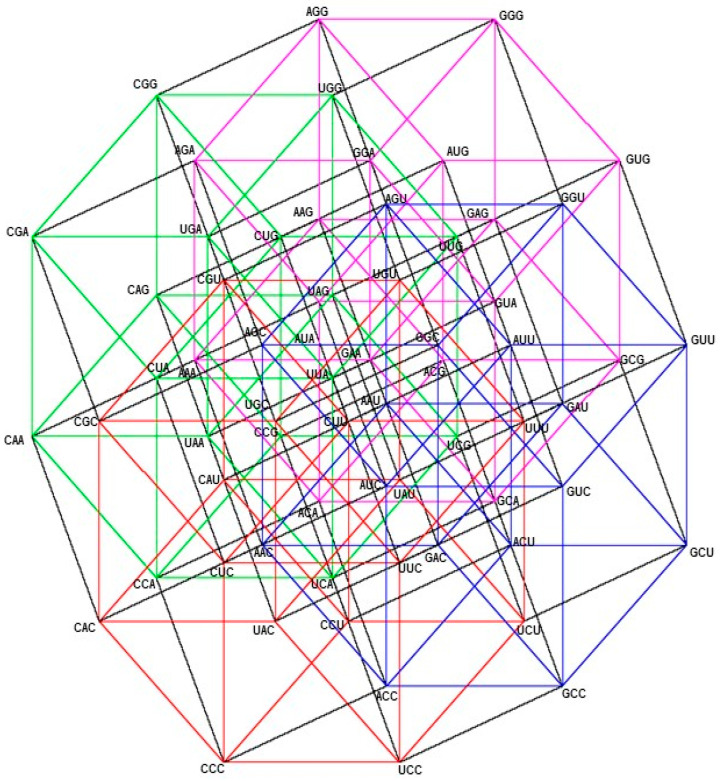
The 6D representation of the Standard Genetic Code.

Note: The n-tuple E is the opposite vertex, in the hypercube ${\left[0,1\right]}^{n},$ of the null vector $O=\left(0,0\ldots 0\right).$ The distance between them is the real number $\sqrt{n}=\left|E\right|,$ which is the diameter of that hypercube. The diameter of a hypercube is the diameter of the hypersphere that circumscribes it-Actually, the hypercube ${\left[-1,1\right]}^{n}$ is the image of ${\left[0,1\right]}^{n}$ under the affine transformation $T_{-E}\circ 2I$ being I the identical automorphism. The new hypercube has its center at the origin $O=\left(0,0\ldots 0\right)=T_{-E}\circ 2I\left(\frac{1}{2},\frac{1}{2},\ldots ,\frac{1}{2}\right)$ of coordinates, and its radius is $\sqrt{n}$ (Figure 6). The affine transformation $T_{-E}\circ 2I$ converts every 0 into −1 and the number 1 into itself. The linear transformation E is a homothetic transformation of ratio 2, which duplicates the size of every n-dimensional Figure. Its only fixed point is the origin O of the coordinates. The affine transformation $T_{-E}\circ 2I$ is also a homothety of ratio 2, whose only fixed point is the point E.

## 4. Discussion

The emergence of the first encoded processing system at the molecular level triggered the phenomenon of life. Coding systems are an inherent property of biological systems. When analyzing the origin and evolution of codes, it becomes clear that the overlap of new codes over earlier/older ones increased the complexity of the previous codes, operating to better adjust and fine-tune them. These interrelated codes started to interact to form a macrocode composed of multiple, overlapping coding systems. Therefore, an information system should be understood as any system capable of implementing the (encoded) connection between natural entities. Life should be understood as an information system that is dependent on a code (open or closed) for its processing but operates independently of other macrocode structures [20].

The distribution of aaRSs in the subcodes is not random. From the putative ancestral symmetric gene to the whole SGC, there is always a symmetrical distribution along the evolution of the genetic code.

Class aaRS I (Class aaRS II) can be converted into Class aaRS II (Class aaRS I) by means of isometric algebraic functions [19]. The fact that enzymes belonging to the two synthetase classes are grossly mirror images of each other (e.g., they approach opposite sides on tRNAs) motivated a phylogenetic analysis that indicated that these proteins were originally coded for by opposite strands of the same gene [15,16,17] in the later stages of the RNA world. This scenario was strengthened experimentally [18]. When the symmetry groups have a structure-preserving one-to-one correspondence, they are considered isomorphic [21].

All synthetases of Class II can be found in the first two 4D-hypercubes (RNY and YNY). Moreover note that Class I and II of aaRSs existed for all 20 amino acids in the Extended RNA codes 1 and 2, before the completion of the SGC. According to the symmetries found in the last step to arrive at the SGC, duplications of Lys, Arg, Glu, Gly, Pro, Leu, Ser, and Phe for the Extended RNA code type 1 and duplications of Gln, Arg, Stop, Trp, Pro, Leu, and Ser for the Extended RNA code type 2 were necessary, resulting in a simpler algebraic structure of the SGC (see Table 1).

The hypercubes consider mono-codonic, di-codonic, three-codonic, tetra-codonic, and hexa-codonic amino acids. In each code, we can observe symmetrical structures in the distribution of aaRSs. We observed that the symmetrical properties of the aaRSs distribution in the SGC are simpler than the ones observed for both Extended RNA codes. In short, there are only 20 aaRSs, one for each amino acid and, respectively, for isoacceptor tRNAs; hence, the aaRS link to the 20 coded amino acids is non-degenerate [3].

Our group-theoretical approach does not explicitly consider how the allocation of aaRSs during the evolution of the genetic code was constrained by the structural and functional properties of the interaction of aaRS, amino acids, and tRNA. Yet it permits us to determine the types of symmetries of aaRSs during the evolution of the SGC. The symmetry groups found in the RNA codes highlight intricacies in the evolution of aaRSs in conjunction with the evolution of the genetic code itself.

We have determined the type of symmetries of aaRSs in each of the 4 subcodes of the Extended RNA code type II. We used the 4D representation of each subcode. The RNY subcode has the dihedral group, with 8 symmetries, 4 rotations, and 4 reflections. The YNY subcode has $Z_{2}$ symmetry with 2 symmetries; the YNR subcode exhibits octahedral amplified symmetry with 48 elements, 24 rotations, and 24 roto-reflections (the octahedral classic group contains only 24 rotations); and the RNR subcode displays the symmetry of the binary group $Z_{2}.$ In each subcode, symmetrical distributions of both Classes of aaRSs were found. Indeed, we proved that for each hypercube, the group of symmetries of Class 1 is the same as the group of symmetries of Class 2. This theorem holds for both 5D hypercubes and for the whole 6D representation of the SGC. Note that the distribution of aaRSs in both Extended RNA code type I and II and the SGC is symmetrical, which is consistent with the notion that the evolution of the two aaRS classes evolved in parallel [21].

The consideration of stop codons in the determination of the type of symmetry of the subcode is not fortuitous. Mitochondrial codes present variations principally in the codons for the stop signals and unicoded amino acids. The mitochondrial genetic codes of yeast, invertebrates, and vertebrates present variations principally in the codons for the stop signals and unicoded amino acids https://www.ncbi.nlm.nih.gov/Taxonomy/Utils/wprintgc.cgi?chapter=tgencodes#SG24 (accessed on 31 July 2023). The stop codons are tricodonic in SGC, tetracodonic in vertebrate mitochondria, and dicodonic in invertebrate and yeast mitochondria.

In computer science, a byte is an octet of 0’s and 1’s, where each bit represents 0 or 1. Hence, in the genetic code, a byte would correspond to a sextuple of 0’s and 1’s, where each of them represents a triplet or codon of the nucleotides $\left\{C,U,A,G\right\}.$ The presence of stop codons converts the genetic code into an algorithm that carries out protein synthesis. This means that the whole process of protein synthesis is carried out by a Turing machine, i.e., by a recursive function. Unlike the Turing machine, the genetic code has the additional property of heritability. In a forthcoming work, we will develop these concepts.

Our work can be regarded as a possible pathway for the distribution of the 2 Classes of aaRSs during the formation of the SGC. According to the model of Rodin-Ohno [15,16,17,18], there was a single gene encoding for two synthetases. They proposed a single anti-parallel complementary strand of a single base-paired nucleic acid molecule. The Rodin-Ohno model divides the table of the genetic code into two classes of aminoacyl-tRNA synthetases (Classes 1 and 2), with recognition from the minor or major groove sides of the tRNA acceptor stem [15,16,17,18]. According to the table of the genetic code, the RO model is almost symmetric. It turns out that the RO model is symmetric in a six-dimensional (6D) hypercube (José et al. 2017). Conversely, using the same automorphisms, the RO model can lead to the SGC. Class aaRS 1 (Class aaRS 2) can be converted into Class aaRS II (Class aaRS I) by means of isometric algebraic functions [19]. Notably, the 6D algebraic model is compatible with both the SGC (based upon the primeval RNY code) and the RO model [19]. Our results have implications in areas such as creating synthetic codes, astrobiology, and computer science. In astrobiology, it provides new insights into the quest for life in the Universe. In computer science, it provides a guideline for the establishment of decision criteria to define what should be considered an artificial life.

## Figures and Tables

**Table 1:** Genetic code: Triplets and amino acids in each of the 4-, 5-, and 6-dimensional hypercubes. aaRS Class 1, one red asterisk *; aaRS Class 2, two blue asterisks **. RNY triplets in gold; YNY triplets in black; YNR triplets in blue; RNR triplets in green.

5D NNY		5D NNR	
4D RNY	4D YNY	4D YNR	4D RNR
**ACC**	**CCC**	**CCA**	**ACA**
Thr **	Pro **	Pro **	Thr **
**ACU**	**CCU**	**CCG**	**ACG**
Thr **	Pro **	Pro **	Thr **
**AUC**	**CUC**	**CUA**	**AUA**
Ile *	Leu *	Leu *	Ile *
**AUU**	**CUU**	**CUG**	**AUG**
Ile *	Leu *	Leu *	Met *
**AAC**	**CAC**	**CAA**	**AAA**
Asn **	His **	Gln *	Lys *
**AAU**	**CAU**	**CAG**	**AAG**
Asn **	His **	Gln *	Lys *
**AGC**	**CGC**	**CGA**	**AGA**
Ser **	Arg *	Arg *	Arg *
**AGU**	**CGU**	**CGG**	**AGG**
Ser **	Arg *	Arg *	Arg *
**GCC**	**UCC**	**UCA**	**GCA**
Ala **	Ser **	Ser **	Ala **
**GCU**	**UCU**	**UCG**	**GCG**
Ala **	Ser **	Ser **	Ala **
**GUC**	**UUC**	**UUA**	**GUA**
Val *	Phe **	Leu *	Val *
**GUU**	**UUU**	**UUG**	**GUG**
Val *	Phe **	Leu *	Val *
**GAC**	**UAC**	**UAA**	**GAA**
Asp **	Tyr *	**Stop**	Glu *
**GAU**	**UAU**	**UAG**	**GAG**
Asp **	Tyr *	**Stop**	Glu *
**GGC**	**UGC**	**UGA**	**GGA**
Gly **	Cys *	**Stop**	Gly **
**GGU**	**UGU**	**UGG**	**GGG**
Gly **	Cys *	Trp *	Gly **

**Table 2:** The Cayley tables of sum and multiplication of the binary set $Z_{2}$.

${⊕}_{2}$	0	1
0	0	1
1	1	0

${⊕}_{2}$	0	1
0	0	0
1	0	1

**Table 3:** Sum module 2 of nucleotide bases.

${⊕}_{2}$	C	U	A	G
C	C	U	A	G
U	U	U	G	A
A	A	G	C	U
G	G	A	U	C

**Table 4:** Classes and subclasses of aminoacyl tRNA synthetases.

Class 1	Class 2
1a{MetRS, ValRS, LeuRS, IleRS, CysRS, ArgRS	2a{SerRS, ThrRS, AlaRS, GlyRS-α2, ProRS, HisRS
1b{GluRS, GlnRS, LysRS	2b{AspRS, AsnRS, LysRS
1c{TyrRS, TrpRS	2c{PheRS, GlyRS-α2β2, SepRS, PylRS

**Table 5:** Summary of symmetry groups of aaRSs for each subcode.

Hypercube	Group of Symmetries
**RNY**	$D_{4}$
**YNY**	ℤ_2_
**YNR**	O_h_
**RNR**	ℤ_2_

## Data Availability

Not applicable.

## Appendix A

Let us consider the orthogonal matrix $A=\left(\begin{array}{l} 1\;0\;0\;0\\ 0\;0\;0\;1\\ 0\;0\;1\;0\\ 0\;-1\;0\;0 \end{array}\right),$ which represents a rotation of angle $\frac{3\pi }{2}$ in the plane $\langle e_{2},e_{4}\rangle .$ It operates over the set of vertexes of the square S in the following way:

$$A:\left(\begin{array}{l} 1\\ -1\\ -1\\ -1 \end{array}\right)\to \left(\begin{array}{l} 1\\ -1\\ -1\\ 1 \end{array}\right)\to \left(\begin{array}{l} 1\\ 1\\ -1\\ 1 \end{array}\right)\to \left(\begin{array}{l} 1\\ 1\\ -1\\ -1 \end{array}\right)\to \left(\begin{array}{l} 1\\ -1\\ -1\\ -1 \end{array}\right)$$

This means that A is an isometry of the square S.

Let us now consider the orthogonal matrix $B=\left(\begin{array}{l} 1\;0\;0\;0\\ 0\;1\;0\;0\\ 0\;0\;1\;0\\ 0\;0\;0\;-1 \end{array}\right),$ which represents a reflection through the 3-dimensional hyperplane $\langle e_{1},e_{2},e_{3}\rangle ,$ that reverses the fourth axis $R_{e_{4}}$ It operates over the set of vertexes of the square S in the following way:

$$B:\left(\begin{array}{l} 1\\ -1\\ -1\\ -1 \end{array}\right)\to \left(\begin{array}{l} 1\\ -1\\ -1\\ 1 \end{array}\right)\to \left(\begin{array}{l} 1\\ -1\\ -1\\ -1 \end{array}\right)$$

$$B:\left(\begin{array}{l} 1\\ 1\\ -1\\ 1 \end{array}\right)\to \left(\begin{array}{l} 1\\ 1\\ -1\\ -1 \end{array}\right)\to \left(\begin{array}{l} 1\\ 1\\ -1\\ 1 \end{array}\right).$$

This means that B is an isometry of the square S. It is not difficult to verify the following equalities, that occur between the matrixes A and B: $A^{4}=B^{2}=I;\;\mathrm{BA}=A^{3}B.$

They are the defining relations of the dihedral group $D_{4},$ the group of all the symmetries of a square.

Now, we will see which is the action of the dihedral group $D_{4},$ generated by the matrixes A and B, over the set $V_{1}$ of the 12 quadruplets that represent the synthetases of Class 1.

The action of A over the set $V_{1}$ is,

$$A:u_{1}=\left(\begin{array}{l} -1\\ -1\\ -1\\ -1 \end{array}\right)\to u_{2}=\left(\begin{array}{l} -1\\ -1\\ -1\\ 1 \end{array}\right)\to u_{3}=\left(\begin{array}{l} -1\\ 1\\ -1\\ 1 \end{array}\right)\to u_{4}=\left(\begin{array}{l} -1\\ 1\\ -1\\ -1 \end{array}\right)\to u_{1}=\left(\begin{array}{l} -1\\ -1\\ -1\\ -1 \end{array}\right)$$

$$u_{5}=\left(\begin{array}{l} -1\\ -1\\ 1\\ -1 \end{array}\right)\to u_{6}=\left(\begin{array}{l} -1\\ -1\\ 1\\ 1 \end{array}\right)\to u_{7}=\left(\begin{array}{l} -1\\ 1\\ 1\\ 1 \end{array}\right)\to u_{8}=\left(\begin{array}{l} -1\\ 1\\ 1\\ -1 \end{array}\right)\to u_{5}=\left(\begin{array}{l} -1\\ -1\\ 1\\ -1 \end{array}\right)$$

$$u_{9}=\left(\begin{array}{l} 1\\ -1\\ 1\\ -1 \end{array}\right)\to u_{10}=\left(\begin{array}{l} 1\\ -1\\ 1\\ 1 \end{array}\right)\to u_{11}=\left(\begin{array}{l} 1\\ 1\\ 1\\ 1 \end{array}\right)\to u_{12}=\left(\begin{array}{l} 1\\ 1\\ 1\\ -1 \end{array}\right)\to u_{9}=\left(\begin{array}{l} 1\\ -1\\ 1\\ -1 \end{array}\right)$$

It is seen that A is an isometry of the set of synthetases of Class 1.

The action of B over the set $V_{1}$ is:

$$B:u_{1}=\left(\begin{array}{l} -1\\ -1\\ -1\\ -1 \end{array}\right)↔u_{2}=\left(\begin{array}{l} -1\\ -1\\ -1\\ 1 \end{array}\right)\;u_{3}=\left(\begin{array}{l} -1\\ 1\\ -1\\ 1 \end{array}\right)↔u_{4}=\left(\begin{array}{l} -1\\ 1\\ -1\\ -1 \end{array}\right)$$

$$u_{5}=\left(\begin{array}{l} -1\\ -1\\ 1\\ -1 \end{array}\right)↔u_{6}=\left(\begin{array}{l} -1\\ -1\\ 1\\ 1 \end{array}\right)\;u_{7}=\left(\begin{array}{l} -1\\ 1\\ 1\\ 1 \end{array}\right)↔u_{8}=\left(\begin{array}{l} -1\\ 1\\ 1\\ -1 \end{array}\right)$$

$$u_{9}=\left(\begin{array}{l} 1\\ -1\\ 1\\ -1 \end{array}\right)↔u_{10}=\left(\begin{array}{l} 1\\ -1\\ 1\\ 1 \end{array}\right)\;u_{11}=\left(\begin{array}{l} 1\\ 1\\ 1\\ 1 \end{array}\right)↔u_{12}=\left(\begin{array}{l} 1\\ 1\\ 1\\ -1 \end{array}\right)$$

Note that B is an isometry of the set of synthetases of Class 1.

We see that B this is a symmetry of the set $V_{1}$ vertexes of Class 1 synthetases. As A and B are symmetries of the Class 1 of synthetases, this result confirms the assertion of Theorem 1, where it was proved that both classes have the same group of symmetries.

## Appendix B

The group of symmetries of both classes of synthetases is the binary group $\left(Z_{2},{⊕}_{2}\right).$

Let us consider the orthogonal matrix $A=\left(\begin{array}{llll} 0 & 0 & 1 & 0\\ 0 & -1 & 0 & 0\\ 1 & 0 & 0 & 0\\ 0 & 0 & 0 & 1 \end{array}\right),$ which represents a rotation of angle $\pi =180^{\circ }$ around the vectorial plane generated by the vectors $e_{4}$ and $e_{1}+e_{3}+e_{4}=\left(1,0,1,1\right),$ which fixes the point $e_{4}$ changes the sign the vector $e_{2}$ and interchanges the vectors $e_{1}$ and $e_{3}.$

Let us now see what the action of the rotation A over the set $V_{1}$ is:

$$A:v_{1}=\left(\begin{array}{l} 1\\ 1\\ -1\\ -1 \end{array}\right)\to v_{7}=\left(\begin{array}{l} -1\\ -1\\ 1\\ -1 \end{array}\right)$$

$$A:v_{2}=\left(\begin{array}{l} 1\\ 1\\ -1\\ 1 \end{array}\right)\to v_{8}=\left(\begin{array}{l} -1\\ -1\\ 1\\ 1 \end{array}\right)$$

$$A:v_{3}=\left(\begin{array}{l} -1\\ 1\\ -1\\ -1 \end{array}\right)\to v_{5}=\left(\begin{array}{l} -1\\ -1\\ -1\\ -1 \end{array}\right)$$

$$A:v_{4}=\left(\begin{array}{l} -1\\ 1\\ -1\\ 1 \end{array}\right)\to v_{6}=\left(\begin{array}{l} -1\\ -1\\ -1\\ 1 \end{array}\right)$$

$$A:v_{5}=\left(\begin{array}{l} -1\\ -1\\ -1\\ -1 \end{array}\right)\to v_{3}=\left(\begin{array}{l} -1\\ 1\\ -1\\ -1 \end{array}\right)$$

$$A:v_{6}=\left(\begin{array}{l} -1\\ -1\\ -1\\ 1 \end{array}\right)\to v_{4}=\left(\begin{array}{l} -1\\ 1\\ -1\\ 1 \end{array}\right)$$

$$A:v_{7}=\left(\begin{array}{l} -1\\ -1\\ 1\\ -1 \end{array}\right)\to v_{1}=\left(\begin{array}{l} 1\\ 1\\ -1\\ -1 \end{array}\right)$$

$$A:v_{8}=\left(\begin{array}{l} -1\\ -1\\ 1\\ 1 \end{array}\right)\to v_{2}=\left(\begin{array}{l} 1\\ 1\\ -1\\ 1 \end{array}\right)$$

It is seen that the rotation A is a symmetry of the set $V_{1}.$

Next, we will see the action of A over the set of vertexes $V_{1}:$

$$A:u_{1}=\left(\begin{array}{l} 1\\ -1\\ -1\\ -1 \end{array}\right)\to u_{7}=\left(\begin{array}{l} -1\\ 1\\ 1\\ -1 \end{array}\right)$$

$$A:u_{2}=\left(\begin{array}{l} 1\\ -1\\ -1\\ 1 \end{array}\right)\to u_{8}=\left(\begin{array}{l} -1\\ 1\\ 1\\ 1 \end{array}\right)$$

$$A:u_{3}=\left(\begin{array}{l} 1\\ -1\\ 1\\ -1 \end{array}\right)\to u_{5}=\left(\begin{array}{l} 1\\ 1\\ 1\\ -1 \end{array}\right)$$

$$A:u_{4}=\left(\begin{array}{l} 1\\ -1\\ 1\\ 1 \end{array}\right)\to u_{6}=\left(\begin{array}{l} 1\\ 1\\ 1\\ 1 \end{array}\right)$$

$$A:u_{5}=\left(\begin{array}{l} 1\\ 1\\ 1\\ -1 \end{array}\right)\to u_{3}=\left(\begin{array}{l} 1\\ -1\\ 1\\ -1 \end{array}\right)$$

$$A:u_{6}=\left(\begin{array}{l} 1\\ 1\\ 1\\ 1 \end{array}\right)\to u_{4}=\left(\begin{array}{l} 1\\ -1\\ 1\\ 1 \end{array}\right)$$

$$A:u_{7}=\left(\begin{array}{l} -1\\ 1\\ 1\\ -1 \end{array}\right)\to u_{1}=\left(\begin{array}{l} 1\\ -1\\ -1\\ -1 \end{array}\right)$$

$$A:u_{8}=\left(\begin{array}{l} -1\\ 1\\ 1\\ 1 \end{array}\right)\to u_{2}=\left(\begin{array}{l} 1\\ -1\\ -1\\ 1 \end{array}\right)$$

Notice that rotation A is a symmetry of the set $V_{2}.$

The rotation A of an angle $\pi =180^{\circ }$ is an element of order two. Then, the group of symmetries of the classes of synthetases in the hypercube YNY contains the group generated by the rotation A, which is isomorphic to the binary group $\left(Z_{2},{⊕}_{2}\right).$

The isometric transformation A is the only symmetry of the sets, $V_{1}$ and $V_{2}$ in the hypercube YNY. Then, the group of symmetries of both classes of synthetases is the binary group $\left(Z_{2},{⊕}_{2}\right).$

## Appendix C

The group of symmetries of classes of synthetases in the hypercube YNR is the extended octahedral group $\left(O_{h},\circ \right).$

Let us consider the translation $T_{e3}$ associated to the point $e_{3}=\left(0,0,1,0\right).$ Applying it to the set $V_{1}$ we obtain:

$$T_{e3}(V_{1})=U_{1}=\left\{\left(-1,-1,0,-1\right),\left(-1,-1,0,1\right),\left(-1,1,0,1\right),\left(-1,1,0,-1\right),\left(1,-1,0,-1\right),\left(1,-1,0,1\right),\left(1,1,0,1\right),\left(1,1,0,-1\right)\right\},$$

which is the set of vertexes of another cube, with center in the origin O=(0,0,0,0) of coordinates.

Analogously, applying to the set $V_{1}$ the translation $T_{-e_{3}}$ we obtain:

$$T_{-e_{3}}(V_{2})=U_{2}=\left\{\left(-1,-1,0,-1\right),\left(-1,-1,0,1\right),\left(-1,1,0,1\right)\left(-1,1,0,-1\right),\left(1,-1,0,-1\right),\left(1,-1,0,1\right),\left(1,1,0,1\right),\left(1,1,0,1\right)\right\},$$

which is the same set $U_{1},$ of vertexes of a cube with center in the origin O=(0,0,0,0) of coordinates.

Let us now consider the orthogonal matrices:

$$A=\left(\begin{array}{llll} 1 & 0 & 0 & 0\\ 0 & 1 & 0 & 0\\ 0 & 0 & -1 & 0\\ 0 & 0 & 0 & -1 \end{array}\right),\;B=\left(\begin{array}{llll} 1 & 0 & 0 & 0\\ 0 & -1 & 0 & 0\\ 0 & 0 & 1 & 0\\ 0 & 0 & 1 & -1 \end{array}\right)$$

$$C=\left(\begin{array}{llll} 1 & 0 & 0 & 0\\ 0 & 0 & 0 & 1\\ 0 & 1 & 0 & 0\\ 0 & 0 & 1 & 0 \end{array}\right),\;D=\left(\begin{array}{llll} 1 & 0 & 0 & 0\\ 0 & 0 & \;1 & 0\\ 0 & 1 & 0 & 0\\ 0 & 0 & 0 & -1 \end{array}\right)$$

which represent rotations, A,B and D of angles π radians and C of angle $\frac{2\pi }{3}$ radians. They satisfy the relations: $A^{2}=B^{2}=C^{3}=D^{2}=I,$

$$\mathrm{BA}=AB,\;CA=BC,\;CB=ABC,\;DA=BD,\;DB=AD,\;DC=AC^{2}D.$$

that are the defining relations of the octahedral group, $\left(O,\circ \right),$ which is the group of the rotational symmetries of a cube or regular hexahedron. It is also the group of rotational symmetries of a regular octahedron, which is the dual figure of the regular hexahedron. Adding to the set of rotational matrixes A,B,C and D, the matrix

$$R=\left(\begin{array}{llll} 1 & 0 & 0 & 0\\ 0 & 1 & 0 & 0\\ 0 & 0 & 1 & 0\\ 0 & 0 & 0 & -1 \end{array}\right),$$

which represents a reflection through the three-dimensional subspace XYZ, generated by the unitary vectors $e_{1},e_{2},e_{3},$ that reverses the fourth axis $\mathbb{R}e_{4},$ the amplified octahedral group $\left(O_{h},\circ \right)$ is obtained, where $O_{h}=O\cup \left(O\circ R\right).$

We invite the lector to verify that these rotations, and the reflection R, are symmetries of the sets $V_{1}$ and $V_{2}$ of the vertexes of Class 1 and Class 2, respectively.

From this, it follows that the group of symmetries of classes of synthetases in the hypercube YNR is the extended octahedral group $\left(O_{h},o\right).$

## Appendix D

The reflection R is the only symmetry of both Classes 1 and 2.

Now, consider the matrix $R=\left(\begin{array}{llll} 1 & 0 & 0 & 0\\ 0 & 1 & 0 & 0\\ 0 & 0 & 1 & 0\\ 0 & 0 & 0 & -1 \end{array}\right),$ which represents the same reflection of Section 2.4.

Through the hyperplane XYZ, that reverses the axis $\mathbb{R}e_{4}.$ This reflection changes the sign of the fourth component of each quadruple, and it is easy to notice that it produces the following interchange of vectors in the Class I of synthetases:

$$v_{1}↔v_{2},v_{3}↔v_{4},v_{5}↔v_{6}$$

Hence, R is a symmetry in the Class I of synthetases in the hypercube RNR.

By Theorem 1, it should also be a symmetry of the set $V_{2}$ of vertices in Class 2. However, it is straightforward to verify that it produces the interchange of vectors:

$$u_{1}↔u_{2},u_{3}↔u_{4},u_{5}u_{6},u_{7}↔u_{8},u_{9}↔u_{10}.$$

Then, R is also a symmetry of Class 2.

It is not difficult to notice that the reflection R is the only symmetry in both Classes 1 and 2. As it is an element of order two, the group $\left(\left\{R,I\right\}\circ \right),$ isomorphic to the Abelian group $\left(Z_{2},{⊕}_{2}\right)$ is, as in the case of Part II, the group of symmetries of the classes of synthetases in the hypercube RNR.

## Appendix E

Mathematical background

The Euclidean n-dimensional ℝ− space, $\left({\mathbb{R}}^{n},+,\times \right),$ being $\left(\mathbb{R},+,\times \right)$ the field of real numbers, has a basis $\left(e_{1},e_{2},\ldots ,e_{n}\right),$ the so-called canonical basis, where, for each $i\in \left\{1,2,\ldots n\right\},$$e_{i}=\left(0,0,\ldots 1,\ldots ,0\right),$ with 1 is in the place i, and 0 is in the remainders.

**A remarkable observation**: The n-dimensional vector space $\left({\mathbb{R}}^{n},+,\times \right)$ is a generalization of the ordinary 3-dimensional ℝ−space, $\left({\mathbb{R}}^{3},+,\times \right),$ where every triplet $\left(x_{1},x_{2},x_{3}\right)$ represents a point P of the space, endowed with a system of axis X,Y and Z, pairwise orthogonal or perpendicular, intercepted in a common point denoted as O and called the origin of coordinates, represented by the triplet (0,0,0). Usually, every point P is identified with the triplet $\left(x_{1},x_{2},x_{3}\right)$ that represents it, as if they were the same object. So, the equality $P=\left(x_{1},x_{2},x_{3}\right)$ is admitted. The axes are right lines defined as $X=\mathbb{R}e_{1}=\left\{\left(x_{1},0,0\right)\left|x_{1}\in \mathbb{R}\right.\right\},$ $Y=\mathbb{R}e_{2}=\left\{\left(0,x_{2},0\right)\left|x_{2}\in \mathbb{R}\right.\right\}$, $Z=\mathbb{R}e_{3}=\left\{\left(0,0,x_{3}\right)\left|x_{3}\in \mathbb{R}\right.\right\}$, The addition of two points $P=\left(x_{1},x_{2},x_{3}\right)$ and $Q=\left(y_{1},y_{2},y_{3}\right)$ is defined as $P+Q=\left(x_{1}+y_{1},x_{2}+y_{2},x_{3}+y_{3}\right).$ This operation, therefore defined, is associative, has a neutral element, O, and for every point $P=\left(x_{1},x_{2},x_{3}\right)$ its additive inverse $-P=\left(-x_{1},-x_{2},-x_{3}\right)$ exists, such that $P+\left(-P\right)=O.$ Then, the ordered pair $\left({\mathbb{R}}^{3},+\right)$ is a group. As the addition + is also commutative, it is an Abelian group. The product of a real number α∈ℝ by a point $P=\left(x_{1},x_{2},x_{3}\right)$ is defined as $\alpha \times P=\left(\alpha x_{1},\alpha x_{2},\alpha x_{3}\right).$ This external operation of a real number by a point has the following algebraic properties:

1. $\alpha \times \left(\beta \times P\right)=\left(\alpha \times \beta \right)\times P$ for $\alpha ,\beta \in \mathbb{R},\;P\in {\mathbb{R}}^{3}$ (Mixed associativity).
2. 1×P=P, for every $P\in {\mathbb{R}}^{3}$ (Existence of an external neutral element).
3. $\alpha \times \left(P+Q\right)=\alpha \times P+\alpha \times Q,$ for all $\alpha \in \mathbb{R},\;P,Q\in {\mathbb{R}}^{3}$ (Distributivity of the product with respect to the addition of points, also called distributivity at the left).
4. $\left(\alpha +\beta \right)\times P=\alpha \times P+\beta \times P$ for all $\alpha ,\beta \in \mathbb{R},\;P\in {\mathbb{R}}^{3}$(Distributivity of the product with respect to the addition of numbers, also called distributivity at the right.)

The condition of the Abelian group for the addition and the four properties of the external operation completes the structure of a vector space over the field $\left(\mathbb{R},+,\times \right)$ of the real numbers, in the set ${\mathbb{R}}^{3}$ of all the points of the space.

**The concept of vector:** Given two different points $P,Q\in {\mathbb{R}}^{3}$ we call the vector of origin P and extreme Q to the oriented segment of the right line that joins P with Q. It is denoted as $\vec{\mathrm{PQ}}.$ We define the addition of two vectors $\vec{\mathrm{PQ}}$ and PR with common origin P as the vector $\vec{\mathrm{PS}}$ such that S=R+Q-P. If the points R,Q and P are not collinear, the four points are the vertices of a plane parallelogram. It means that the lines $r\left(P,Q\right)$ and $r\left(R,S\right)$ are parallel. If P=O, the null point $\left\{0,0,0\right\},$ the function: $f:P\to \vec{\mathrm{PO}},$ where for P=O, $\vec{\mathrm{OO}}=0$ is defined as a vector whose initial and final points coincide, is a bijective function between the set ${\mathbb{R}}^{3}$ of all the triplets or points of the space, and the set V of all the vectors with origin O. This correspondence induces over the set V the operations + and × of addition and product of numbers by vectors and V is endowed the structure of a vector space over the field $\left\{\mathbb{R},+,\times \right\}$ of the real numbers.

Euclidean norm

The Euclidean norm, module, or length, of a vector $v=\left(x_{1},x_{2},\ldots x_{n}\right)$ is defined as $\left|v\right|=\sqrt{x_{1}^{2}+x_{2}^{2}+\ldots x_{2}^{n}},$ the non-negative square root of the sum of the squares of its n components.

Main properties of the Euclidean norm

(N1) For every $v,\;\left|v\right|\geq 0,$ and it is =0 if, and only if, v=0, the null vector O=(0,0,0…0), (Positivity of the norms for all the non-null vectors)

(N2) For $v\in {\mathbb{R}}^{n}$ and α∈ℝ, $\left|\alpha \times v\right|=\left|\alpha \right|\left|v\right|.$ (The norm $\left|\alpha \right|,$ module, or absolute value, of the real number α≠0 is the positive of the couple $\left\{\alpha ,-\alpha \right\},$ and for $\alpha =0,\;\left|\alpha \right|=0.$

From N_2_ we have that $\left|-v\right|=\left|v\right|$ for every $v\in {\mathbb{R}}^{n}.$

(N3) For all $u,v\in {\mathbb{R}}^{n},$ $\left|u+v\right|\leq \left|u\right|+\left|v\right|$ (Triangle inequality).

The Euclidean Distance between vectors

For $u,v\in {\mathbb{R}}^{n},$ the Euclidean distance $d\left(u,v\right)$ between their extremes, is defined as $\left|u-v.\right|$

Main properties of the Euclidean distance

(D1) For every v, $d\left(v,O\right)=\left|v\right|\geq 0,$ being O the null vector $O=\left(0,0,\ldots 0\right).$ (Positivity of the distance to the origin for all the final points of the non-null vectors).

(D2) For all $u,v\in {\mathbb{R}}^{n},$ $d\left(u,v\right)=d\left(v,u\right)$ (Symmetry).

(D3) For all $u,v\in {\mathbb{R}}^{n},$ $d\left(u,w\right)\leq d\left(u,v\right)+d\left(v,w\right)$ (Triangle inequality).

With this distance the set ${\mathbb{R}}^{n},$ of vectors, is a metric space and the vector space $\left({\mathbb{R}}^{n},+,*\right)$ is called the n-dimensional Euclidean Vector Space.

Linear transformations

A function $f:{\mathbb{R}}^{n}\to {\mathbb{R}}^{n}$ is called a linear transformation, or linear endomorphism, if it has the following property:

For all $u,v\in {\mathbb{R}}^{n},$ and α,β∈ℝ, the equality $f\left(\alpha *u+\beta *v\right)\;=\;\alpha *f\left(u\right)+\beta *f\left(v\right)$ holds.

Note: The latter condition is equivalent to the two conditions:

(L1) $f\left(u+v\right)=f\left(u\right)+f\left(v\right),$ for all $u,v\in {\mathbb{R}}^{n}.$ (This means that f is a group homomorphism of the additive group $\left({\mathbb{R}}^{n},+\right)$ onto itself).

(L2) $f\left(\alpha \times u\right)=\alpha \times f\left(u\right)$ for all $u\in {\mathbb{R}}^{n}$ and α∈ℝ.

If the function f is bijective, the linear transformation is a linear isomorphism, and it is called a linear automorphism of the vector space.

Matrix representation of a linear transformation

For every endomorphism f there is a unique square matrix $A=\left(a_{\mathrm{ij}}\right),$ such that for every j,

$f\left(e_{j}\right)=\left(a_{1j},a_{2j},\ldots a_{\mathrm{nj}}\right),$ being $\left(\begin{array}{l} a_{1j}\\ a_{2j}\\ \ldots \\ a_{\mathrm{nj}} \end{array}\right)$ the j-th column of A. Then, A is a matrix whose columns are the column matrixes associated with the vector images of the canonical unitary vectors. It is called the matrix of f with respect to the canonical basis. It will be denoted as $A=\left[f\right].$ In the case of an automorphism, the matrix A is a non-singular or invertible matrix; hence, its determinant is different from 0.

For every vector $v=\left(x_{1},x_{2},\ldots x_{n}\right)\in {\mathbb{R}}^{n}$ the column matrix $\left[V\right]=\left[\begin{array}{l} x_{1}\\ x_{2}\\ .\\ .\\ x_{n} \end{array}\right]$ is taken as the matrix representation of V. It is well known that the matrix representation of the image vector f(V) is the matrix product $\left[f\right]\left[V\right].$ Conversely, every square n×n matrix A defines an endomorphism f, whose matrix representation $\left[f\right]$ is A.

Translations

For any vector u the translation defined by u, is the function $T_{u}:{\mathbb{R}}^{n}\to {\mathbb{R}}^{n},$ such that, for all v, $T_{u}(v)=v+u.$ For every $u,T_{u}$ is a bijective function that preserves the norm of each vector and the distance between different vectors of the space. Hence, it is an isometry of the Euclidean vector space. For two translations $T_{u}$ and $T_{v}$ the composition $T_{u}\circ T_{v}$ is equal to the translations $T_{u+v}.$

The correspondence $u↔T_{u}$ between ${\mathbb{R}}^{n}$ and the set T, of all the translations, is a bijective function, that induces over T the structure of a n-dimensional vector space, isomorphic to the Euclidean n-space $\left({\mathbb{R}}^{n},+,\times \right).$

The concept of affine transformation

An affine transformation is a function that is a composition $T_{u}\circ f$ of a linear endomorphism f, followed by a translation $T_{u}.$ It acts on every vector v as $\left(T_{u}\circ f\right)\left(v\right)=f(v)+u.$ The affine transformation $T_{u}\circ f$ is bijective if, and only if, its linear part f is an automorphism. The set of all the affine transformations of the vector space $\left({\mathbb{R}}^{n},+,\times \right)$ will be denoted as $A_{\mathrm{ff}}\left({\mathbb{R}}^{n}\right).$ The composition $\left(T_{u}\circ f\right)\circ \left(T_{v}\circ g\right)$ of two affine transformations $T_{u}\circ f$ and $T_{v}\circ g$ acts over a vector w in the form:

$$w\mapsto \left(T_{u}\circ f\right)\left(g\left(w\right)+v\right)=f\left(g\left(w\right)+v\right)+u=\left(f\circ g\right)\left(w\right)+f\left(v\right)+u=\left(T_{f\left(v\right)+v}\circ \left(f\circ g\right)\right)\left(w\right)$$

Then, the composition is the affine transformation $\left(T_{f\left(v\right)+v}\circ \left(f\circ g\right)\right).$ Hence, we have equality $\left(T_{u}\circ f\right)\circ \left(T_{v}\circ g\right)=T_{f\left(v\right)+v}\circ \left(f\circ g\right).$

We see that the composition of two affine transformations is an affine transformation. Then, the composition is an inner operation in the set $A_{\mathrm{ff}}\left({\mathbb{R}}^{n}\right).$ As the operation is associative and has a neutral element, the identity function I, it is the affine transformation $T_{\circ }\circ I,$ the ordered pair $\left(A_{\mathrm{ff}}\left({\mathbb{R}}^{n}\right),\circ \right)$ is a monoid or semigroup with a neutral element.

Matrix representation

The matrix representation of the affine transformation $T_{u}\circ f$ is given by the composition $T_{\left[u\right]}\circ A,$ being A the matrix of f, which acts over the matrix $\left[v\right]$ of a vector v as $\left[v\right]\to A\left[v\right]+\left[u\right].$

The conjugated of a translation by a linear automorphism

The conjugated $f\circ T_{v}\circ f^{-1}$ acts over any vector w as $w\to \left(f\circ T_{v}\circ f^{-1}\right)\left(w\right)=f\left(f^{-1}(w)+v\right)=w+f\left(v\right)=T_{{}_{}(v)}\left(w\right).$ Then $f\circ T_{v}\circ f^{-1}=T_{f\left(v\right)}.$ The latter means that the group of translations $\left(T,\circ \right)$ is a normal subgroup of the group $\left(A\left({\mathbb{R}}^{n}\right),\circ \right)$ being $A\left({\mathbb{R}}^{n}\right)=T\circ \mathrm{Aut}\left({\mathbb{R}}^{n}\right)$ the set of all the invertible affine transformations, and $\left(\mathrm{Aut}\left({\mathbb{R}}^{n}\right),\circ \right)$ the group of all the linear automorphisms of the space $\left({\mathbb{R}}^{n},+,\times \right).$ The group $\left(A\left({\mathbb{R}}^{n}\right),\circ \right),$ that is called the affine group of the space $\left({\mathbb{R}}^{n},+,\times \right).$ is a subgroup of the group of all the bijective transformation of the set ${\mathbb{R}}^{n}.$ It contains as a normal subgroup, the group $\left(T,\circ \right)$ of all the translations. In fact, it is the semidirect product of the group $\left(T,\circ \right)$ with the group $\left(\mathrm{Aut}\left({\mathbb{R}}^{n}\right),\circ \right)$ of all the linear automorphisms. (A group $\left(G,*\right)$ is semidirect product of its subgroups $\left(K,*\right)$ and $\left(H,*\right)$ if G=K*H and $\left(K,*\right)$ is a normal subgroup of $\left(G,*\right)).$ The semidirect product will be denoted as $\left(K⊲H,*\right).$ Then, $\left(A_{\mathrm{ff}}\left({\mathbb{R}}^{n}\right),\circ \right)=\left(T⊲\mathrm{Aut}\left({\mathbb{R}}^{n}\right),\circ \right).$ The affine group $\left(A\left({\mathbb{R}}^{n}\right),\circ \right)$ is the group of invertible elements of the monoid $\left(A_{\mathrm{ff}}\left({\mathbb{R}}^{n}\right),\circ \right).$

The n-dimensional binary hypercube

The binary vector space $\left(Z_{2}^{n},{⊕}_{2},*\right),$ vector space over the binary field $\left(Z_{2},{⊕}_{2},{⊕}_{2}\right),$ is also called the n-dimensional binary hypercube. Its set of vertexes coincides with the set ${\left\{0,1\right\}}^{n}$ of the $2^{n}$ n-tuples of zeros and ones. This set is a subset of the set $Z^{n}$ of all the n-tuples of the whole numbers. The set $Z^{n}$ determines the Abelian group $\left(Z^{n},+\right),$ subgroup of the additive group $\left({\mathbb{R}}^{n},+\right)$ of the n-dimensional Euclidean n-space $\left({\mathbb{R}}^{n},+,\times \right).$

The n-dimensional hypercube, whose vertexes are the elements of the set ${\left\{0,1\right\}}^{n}$ is defined as the convex closure ${\left[0,1\right]}^{n}$ of the set ${\left\{0,1\right\}}^{n}.$ Here, $\left[0,1\right]$ denotes the closed interval of the real numbers between 0 and 1. The convex closure of a set is the minimal convex set that contains it.

A convex set is a set $C\subseteq {\mathbb{R}}^{n}$ that, for two different elements a and b of C, the segment that joins them is entirely contained in C.

The center of the hypercube ${\left[0,1\right]}^{n}$ is the point $C=\left\{\frac{1}{2}.\frac{1}{2},\ldots ,\frac{1}{2}\right\}$ obtained by the sum of the $2^{n}$ vertexes divided by $2^{n}.$ That is so because, for every place i of the n-tuples, $2^{n-1}$ of them, that is, the half, have 1, and the other $2^{n-1}$ have 0. Then, for each place i, the quotient is $\frac{2^{n-1}}{2^{n}}=\frac{1}{2}.$

**A metric in the hypercube** $Z_{2}^{6}$

**The Hamming Norm and the Hamming Distance defined in** $Z_{2}^{6}$

**The Hamming Norm:** The Hamming Norm $\left|u\right|$ of a sextuple $u=\left(a_{1},a_{2},a_{3},a_{4},a_{5},a_{6}\right)$ is defined as the sum ${\sum a}_{i}$ in Z, of its components $a_{i}.$ It is equal to the number of ones it has. The only unitary sextuples, that is, of norm equal to 1, are those of the canonical basis.

**The Hamming distance.** The Hamming distance $H\left(u,v\right)$ between two sextuples $u=\left(a_{1},a_{2},a_{3},a_{4},a_{5},a_{6}\right)$ and $v=\left(b_{1},b_{2},b_{3},b_{4},b_{5},b_{6}\right)$ is defined as the norm $\left|u{⊕}_{2}v\right|$ of their module 2 addition. It is equal to the number of places where they differ. It is not difficult to prove that the distance so defined is actually a distance, in the mathematical sense. It means that it has the following properties:

(D1) For all u,v, $H\left(u,v\right)\geq 0,$ $H\left(u,v\right)=0\;\Leftrightarrow u=v$ (positivity of the distance between two different sextuples u and v), and nullity of the distance of a sextuple to itself.

(D2) For all u,v, $H\left(u,v\right)=H\left(v,u\right)$ (Symmetry)

(D3) For u,v,w, $H\left(u,w\right)\leq H\left(u,v\right)+H\left(v,w\right)$ (Triangle inequality)

The edges of the hypercube

Two vertexes are said to be neighbors if the distance between them is equal to 1. It means that they differ in only one of their n components. An edge of the hypercube is the segment that joins two neighbor vertexes. This segment has a length equal to 1. For every vertex there are n neighbors. As there are $2^{n}$ vertexes, the product $2^{n}\times n$ would count the total number of edges. However, as every edge has two extremes, it would be counted twice. Then, the actual number of edges is equal to $\frac{2^{n}\times n}{2}=2^{n-1}\times n$. For example, in the ordinary cube, 3-dimensional, the number of edges is $2^{2}\times 3=12$ and, in the 4-dimensional, this number is $2^{3}\times 4=32.$

The faces of the hypercube

Any face of the hypercube is a plane square determined by four vertexes and four edges in such a way that two edges with a common vertex are orthogonal. If we fix n−2 components with values zeros and ones and leave free the other two, the four obtained vertexes are the vertexes of a plane square, then of the face of the hypercube. It may be conducted in $\left(\begin{array}{l} n\\ n-2 \end{array}\right)\times 2^{n-2}$ ways. Hence, the total number of different faces is precisely $\left(\begin{array}{l} n\\ n-2 \end{array}\right)\times 2^{n-2}.$ (The combinatorial number $\left(\begin{array}{l} n\\ k \end{array}\right)=\frac{n!}{k!\left(n-k\right)!}$ is the number of subsets of k elements of a set of n elements, being 0≤k≤n). For example, for n = 3 the number of faces is 3×2=6, and for n = 4 it is 6 × 4 = 24.
